# Rank-based edge reconstruction for scale-free genetic regulatory networks

**DOI:** 10.1186/1471-2105-9-75

**Published:** 2008-01-31

**Authors:** Guanrao Chen, Peter Larsen, Eyad Almasri, Yang Dai

**Affiliations:** 1 Department of Computer Science (MC152), University of Illinois at Chicago, 851 South Morgan Street, Chicago, IL 60607, USA; 2 Core Genomics Laboratory, Research Resource Center (MC937), University of Illinois at Chicago, 835 South Wolcott Avenue, Chicago, IL 60612, USA; 3 Department of Bioengineering (MC063), University of Illinois at Chicago, 851 South Morgan Street, Chicago, IL 60607, USA

## Abstract

**Background:**

The reconstruction of genetic regulatory networks from microarray gene expression data has been a challenging task in bioinformatics. Various approaches to this problem have been proposed, however, they do not take into account the topological characteristics of the targeted networks while reconstructing them.

**Results:**

In this study, an algorithm that explores the scale-free topology of networks was proposed based on the modification of a rank-based algorithm for network reconstruction. The new algorithm was evaluated with the use of both simulated and microarray gene expression data. The results demonstrated that the proposed algorithm outperforms the original rank-based algorithm. In addition, in comparison with the Bayesian Network approach, the results show that the proposed algorithm gives much better recovery of the underlying network when sample size is much smaller relative to the number of genes.

**Conclusion:**

The proposed algorithm is expected to be useful in the reconstruction of biological networks whose degree distributions follow the scale-free topology.

## Background

The reconstruction of genetic regulatory networks based on microarray gene expression data is one of the most challenging tasks in bioinformatics. The genetic regulatory relationship considered here will be restricted to what might be observed in a microarray experiment: a change in the expression of a regulator gene modulates the expression of a target gene mainly via protein-DNA interactions besides other types of interactions, such as protein-protein interaction. Various approaches have been proposed to this problem, such as Boolean Network and Bayesian Network approaches [1-10], differential equations and steady-state models [11-15], and other statistical and probabilistic methods [16-27]. Each method has its own strengths and weakness [28], however, very few has been considered superior to the others mainly because of the intrinsically noisy property of the data, 'the curse of dimensionality', and the unknown 'true' underlying networks. Various scoring metrics and searching heuristics were proposed in [9] within the Bayesian Network (BN) framework. It was shown that a large amount of data is required in order to have a good recovery of the underlying network. This requirement is easily satisfied in a simulated environment; however, it is unlikely to be met for biological applications. Efforts such as incorporating heterogeneous biological data in network reconstruction have been witnessed to improve the accuracy of the networks [29-35].

As pointed out in [36-38], large scale networks, such as the Internet and the scientific collaboration network, show the scale-free property, i.e., the connections or edges in the networks follow the power law distribution. Many biological networks, including transcription regulatory networks, fall into this category. So far there is little research that has explicitly explored this important property to facilitate the learning of genetic networks from gene expression data. One recent study imposed the scale-free constraint on structure in network inference based on the S-system model [39]. They investigated the performance with a simulated small scale time-course data. On the other hand, different mechanisms have been employed to explain the formation of the scale-free property in large scale networks other than biological networks. Most of the suggested models relate to Preferential Attachment [36]. In contrast to modeling network growing, a model with fixed number of nodes and links was proposed recently [40]. By applying local rewiring moves, the network can reach equilibrium states which have the power law degree distribution. Different mechanisms also were proposed to explain specific properties of different types of networks, such as genetic regulatory networks and the World Wide Web [41].

In this study, we proposed a network reconstruction algorithm that takes into account the scale-free network topology based on a modification of the *Symmetric-N *algorithm originally developed in [42]. The *Symmetric-N *algorithm was used to construct co-expressed gene networks which showed scale-free topology. It was also recently incorporated as a major component in their Nearest Neighbor Network algorithm for clustering expression data for generating functionally coherent clusters [43]. Both our modified and the original algorithms were evaluated on simulated data sets and a 102-gene set of microarray gene expression data from a study of the *Saccharomyces cerevisiae *yeast cell cycle [44]. Compared with the original algorithm, the proposed algorithm demonstrated promising capability in recovering the underlying network structure. The results of our algorithm were further compared with a previous study based on the BN approaches [9]. Our algorithm performed much better on the simulated data when the sample size is small compared with the number of variables, as is most of the currently available microarray expression data.

## Results

### Proposed algorithm

Our algorithm is a modification of the algorithm for network construction proposed in [42]. The algorithm in [42] is based on the concept of *N*-nearest-neighbor and consists of two steps. This algorithm (we name it *Symmetric-N*) is presented in the 'Methods' section. In the first step, for each node in the network, all other nodes are sorted according to the magnitude of correlations of gene expression in descending order. These nodes are considered as potential neighbors. In the second step, each pair of nodes is investigated. If they are both in each other's *N *nearest neighbors, a connection between them is made. Otherwise, they are not connected. Here *N *is a prescribed number for the size of neighbors.

By using the *Symmetric-N *algorithm, Agrawal [42] constructed co-expressed gene networks from several published gene expression data sets and found that the gene networks had small-world characteristics and became scale-free when *N *was above certain threshold. It was shown that this algorithm was able to uncover the scale-free topology, however, no analysis was provided on biological relevance of the co-expressed networks in the study. The major characteristic of a scale-free network is that a few nodes with much higher degrees of connections act as the core of the network and other nodes with much fewer connections act as the periphery of the network. In biological networks such as genetic regulatory networks, the transcription factors (TFs) are more likely to regulate multiple target genes and therefore have more connections compared to those non-TFs. On the other hand, the non-TF genes are only regulated by a few TFs. These observations suggest that the sizes of neighbors for the core and periphery nodes should generally not be equal. This phenomenon motivated a modification of the algorithm *Symmetric-N *so that the unequal neighbor sizes of the core and periphery nodes can benefit the network construction. In step 2 of the *Symmetric-N *algorithm, instead of using the same *N *neighbors for all the nodes, a larger number *N*_*C *_is assigned to a core node and a smaller number *N*_*P *_is assigned to a periphery node. If a periphery node is within the *N*_*C *_nearest neighbors of a core node and the core node is within the *N*_*P *_nearest neighbors of the periphery node, then a connection is made between them. Since the ranges of potential neighbors are different for these two types of nodes, the proposed algorithm is named *Asymmetric-N*. Details of the algorithm are presented in the 'Methods' section.

### Computation study

The original algorithm *Symmetric-N *and our modified algorithm *Asymmetric-N *were evaluated with both simulated gene expression data and microarray gene expression data related to yeast cell cycle. Details on the microarray data, the construction of the simulated networks, and their node degree distributions are presented in the 'Methods' section. Two simulated datasets were derived from a 100-node network and a 20-node network, respectively. The underlying scale-free network for the 100-node network has 10 core nodes and 90 periphery nodes. The directions of edges are more likely to be from core nodes to periphery nodes. The 20-node network was constructed in a similar way. The criteria used to evaluate the performance of the algorithms on the simulated data include *recall*, *precision *and *F-Score*. *Recall *is defined as the ratio of the number of true edges found in the reconstructed network to the number of total edges in the underlying network. *Precision *is defined as the ratio of the number of true edges found in the reconstructed network to the number of total edges found in the reconstructed network. *F-Score *is defined as 2*recall*precision/(*recall *+ *precision*).

The microarray data include 102 gene expression temporal profiles observed over 18 time points derived from the yeast cell cycle gene expression data [44]. For this study, the 'true' interactions were derived from the database of Pathway Studio [45] by submitting the list of genes and querying for instances of published interactions between these genes limited to interaction types 'expression' and 'regulation'. One hundred seventy one published interactions were found for this 102-gene set. It should be noted that this is not a so-called 'golden standard' set for a true evaluation of the learning outcome. We report the percentage of the published edges out of the total edges in the reconstructed network, as the criteria used for the simulated datasets would be inappropriate for this microarray dataset because of the unknown or incomplete 'true' network. For the examination of biological relevance of the predicted edges, we report the percentage of edges whose nodes (genes) share a common Gene Ontology (GO) Biological Process (BP) annotation from the Saccharomyces Genome Database (SGD) GO Slim mapper [46]. Generally, two genes or gene products with a common GO BP annotation are considered likely to interact with each other.

In addition, *γ *in *P*(*k*) ~ *k*^*γ *^of the node degree distribution in the constructed network and the fitness of the distribution, measured by the Coefficient of Determination (*R*^2^), were used for the evaluation of the network structure. The parameters *γ *and *R*^2 ^were computed with the *fit*() function in Matlab (see the 'Methods' section for more details of the *fit*() function). Both *F-score *and *R*^2 ^range between 0 and 1. For a good recovery of the network, *F-Score *is expected to be high; *γ *is expected to be close to the *γ *of the underlying network; and *R*^2 ^is expected to be high. For the 100-node network, *γ *= -1.22 and *R*^2 ^= 0.96 for mixed-degree distribution; for the 102-gene network formed with the published interactions from the Pathway Studio, *γ *= -1.22 and *R*^2 ^= 0.93 for mixed-degree distribution.

### Experiment with simulated data

For each underlying network, 10 different sets of gene expression profiles were generated for a fixed number of samples (time points) and results obtained from the algorithms were averaged.

#### The 100-node network

**Figure 1:**
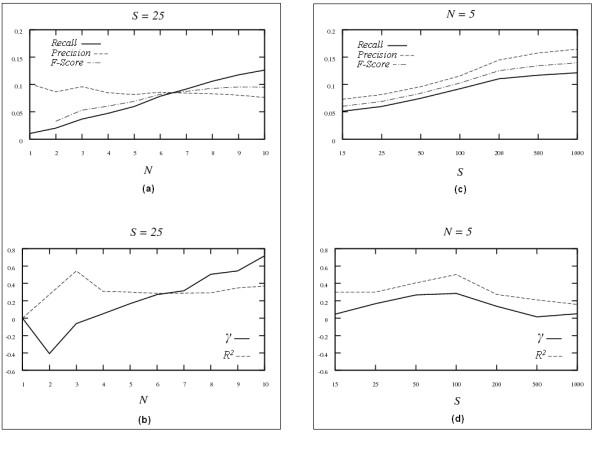
Results for the *Symmetric-N *algorithm with the 100-node simulated network. Panels (a) and (b) show the results when sample size *S *is fixed (*S *= 25) while the number of neighbors *N *is varying. Panels (c) and (d) show the results when *N *is fixed (*N *= 5) while *S *is varying. The upper panels (a) and (c) show the results for *Recall*, *Precision *and *F-Score*. The lower panels (b) and (d) show the results for *γ *and *R*^2^. The parameter pair <*γ*, *R*^2^> for the underlying network structure are <-1.27, 0.96> for in-degree distribution, <-1.61, 0.97> for out-degree distribution, and <-1.22, 0.92> for mixed-degree distribution, respectively.

Figure 1 shows the results obtained from the *Symmetric-N *algorithm on the 100-node network. In panel (a), when the number of samples *S *is fixed at 25, as the number of possible neighbors *N *increases, *recall *increases, *precision *decreases and the *F-Score *first increases (up to *N *= 9) and then decreases (from *N *= 9 to *N *= 10). This is because that as *N *increases, pairs of nodes become more likely to be in each other's neighborhood and thus become more likely to be included. This leads to more true edges in the reconstructed network at the cost of including more edges and decreasing *precision*. Similarly, as *N *becomes larger, *γ *tends to drastically deviate from -1.22, the *γ *of the underlying network, in panel (b); the reconstructed network becomes less scale-free. Here we chose *γ *from the mixed-degree distribution since the reconstructed network is directionless.

In panel (c), while the number of neighbors is fixed (*N *= 5 in this example), increasing the number of samples will generally improve both *recall *and *precision*, therefore also *F-Score*. This result is expected since more observations usually lessen the 'curse of dimensionality', and agrees with the previously published results [9]. The parameter *γ *in panel (d) is still far from the true value (*γ *= -1.22) as the number of samples increases.

**Figure 2:**
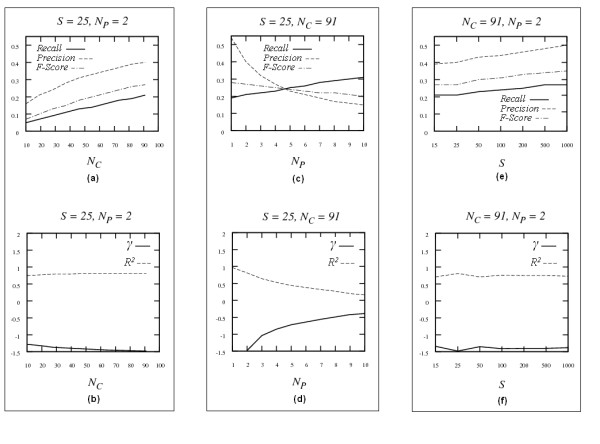
Results for the *Asymmetric-N *algorithm with the 100-node simulated network. Panels (a) and (b) show the results when *S *and *N*_*P *_are fixed (*S *= 25, *N*_*P *_= 2) while *N*_*C *_is varying. Panels (c) and (d) show the results when *S *and *N*_*C *_are fixed (*S *= 25, *N*_*C *_= 91) while *N*_*P *_is varying. Panels (e) and (f) shows the results when *N*_*C *_and *N*_*P *_are fixed (*N*_*C *_= 91, *N*_*P *_= 2) while *S *is varying.

Figure 2 presents the results when applying the *Asymmetric-N *algorithm to the 100-node network. Different from the *Symmetric-N*, the number of neighbors of the core nodes and the periphery nodes were set unequal. Note that there could be different combinations of *N*_*C *_and *N*_*P*_. The values reported in Figure 2 are the ones that achieved the best results according to *F-score *and *γ*. However, the behavior of the algorithms is similar regardless of the choice of values for *N*_*C *_and *N*_*P*_.

In panel (a), we see the trends of *recall*, *precision *and *F-Score *when fixing the number of samples (*S *= 25) and the number of neighbors for the periphery nodes (*N*_*P *_= 2) while varying the number of neighbors for the core nodes (*N*_*C*_). All the three measurements increase as *N*_*C *_increases, which implies that the inclusion of more neighbors for the core nodes generally improves the performance of the algorithm. Similarly, increasing the number of neighbors for the core nodes makes *γ *move toward -1.22 as observed in panel (b). Better results with larger *N*_*C *_for core nodes are consistent with the fact that TFs usually regulate a large number of genes.

In panel (c), *S *and *N*_*C *_are fixed at 25 and 91 respectively, and *N*_*P *_varies. The trends of the three curves show some different patterns compared with those in panel (a): *recall *increases while *precision *decreases and *F-Score *decreases very gently, which means that more false edges are included than true edges when increasing the number of neighbors *N*_*P*_. Similarly, the structure of the network becomes drastically different from the underlying structure as the number of neighbors for the periphery nodes increases (*γ *deviated from -1.22 when *Nc *> 2 in panel (d)). Therefore, this implies periphery nodes should have very few neighbors. This phenomenon is consistent with the fact that non-TF nodes are usually regulated by a few TFs. Similarly, as observed for the *Symmetric-N *algorithm, when fixing *N*_*C *_and *N*_*P*_, the increase of *S *improves the performance of the algorithm for all the three criteria (panel (e)) and the structure of the reconstructed network becomes closer to that of the underlying network (panel (f)).

The performance of *Symmetric-N *and *Asymmetric-N *can be compared by examining Figures 1 and 2. When the number of samples is fixed (*S *= 25) while numbers of neighbors vary, comparing results in panel (a) of Figure 1 with those in panels (a) and (c) of Figure 2, the *Asymmetric-N *algorithm performs much better than the *Symmetric-N *algorithm in terms of *F-Score*, when the number of neighbors for the core nodes is large and the number of neighbors for periphery nodes is small. It is also true for *γ *by comparing panel (b) of Figure 1 with panels (b) and (d) of Figure 2. The same phenomenon is observed when numbers of neighbors are fixed while number of samples changes (comparing panel (c) in Figure 1 with panel (e) in Figure 2, panel (d) in Figure 1 with panel (f) in Figure 2, respectively). The reason is that in the *Symmetric-N *algorithm all the nodes are treated equally while in *Asymmetric-N *algorithm different types of nodes (core and periphery) are distinguished, which reflects biological expectations more closely. Thus the improved performance is expected. In summary, *Asymmetric-N *algorithm outperforms significantly the *Symmetric-N *algorithm proposed in [42].

#### The 20-node network

**Figure 3:**
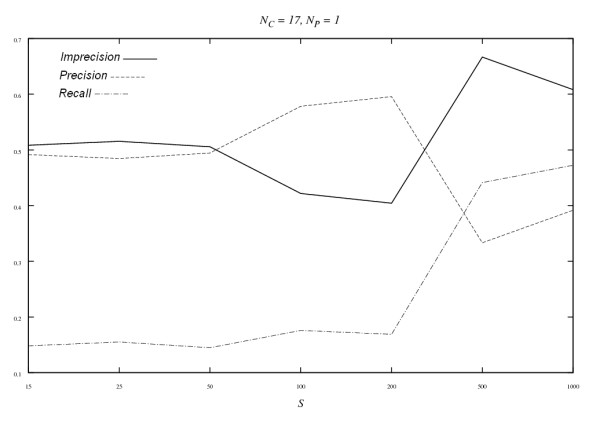
*Recall, precision and imprecision *curves obtained with the *Asymmetric-N *algorithm for the 20-node simulated network when *N*_*C *_and *N*_*P *_are fixed (*N*_*C *_= 17, *N*_*P *_= 1) while *S *is varying. The *imprecision is defined as *1 - *precision*.

We compared the proposed algorithm with some other methods currently used for the reconstruction of transcription regulatory networks. The experiments of Yu *et al*. [9] was selected because our simulated profiles were generated following their procedure, though our networks possess the scale-free property while no structure was assumed in theirs. They applied the BN method to 10 simulated small networks each with 20 nodes, with the number of samples ranging from 25 to 5,000. A *recall-imprecision *curve was used to show the performance when the number of samples increases (*imprecision *= 1 - *precision*). Here, a *recall-imprecision *curve for the *Asymmetric-N *algorithm is drawn for a 20-node network (Figure 3). The largest number of samples is 1,000 in our study. To better appreciate the performance, the *precision *curve (1 - *imprecision*) is shown as well.

It is not surprising that *recall *increases with the number of samples. *Imprecision*, however, increases first and then decreases. It is not clear why this happens and needs further investigation. Fixing at the sample size of *S *= 25, *F-Score *is 0.23, which is better than 0.16 (this number is inferred from Figure 4 in [9]) obtained from the BN method [9]. At larger sample sizes such as 500 and 1,000, the BN approach performs much better. This is reasonable because that the BN method is statistically rigorous and it benefits when more samples are available. However, when only limited samples are available, as is the case in most of the currently available microarray data, our approach may perform better.

### Experiment with the yeast cell cycle microarray data

The results obtained from our *Asymmetric-N *algorithm with different choices of correlation matrices are summarized in Table 1. Results of several combinations of *N*_*C *_and *N*_*P *_are illustrated. The *γ *and *R*^2 ^values, e.g., *γ *= -0.96 and *R*^2 ^= 0.79, deviate far from their counterparts for the underlying network with *γ *= -1.22 and *R*^2 ^= 0.93. This is in contrast to the results that the structural parameters of the reconstructed network are close to their counterparts of the underlying network with the simulation data. The main reason for the inconsistency is that the underlying network in this real dataset is incomplete. The *γ *and *R*^2 ^values for these two networks, namely, the network formed with published interactions and the reconstructed network, might both deviate from those of the real gene interaction network at work in the yeast cell cycle, for which our understanding is still incomplete.

**Table 1:** Results of *Asymmetric-N *on the 102-gene dataset

	**N_C_**	**N_P_**	**#Edges**	**#Published**	**%Published**	**%GO BP**	***γ***	***R*^2^**
**P-P PCC: no time lag**	101	4	142	20	14.08	38.03	-0.69	0.45
**P-P PCC: one time lag**	101	16	185	31	16.76	30.81	-0.96	0.79
**S-S PCC: no time lag**	91	1	60	17	28.33	45.83	-1.27	0.65
**S-S PCC: one time lag**	91	11	155	29	18.71	27.74	-0.89	0.70

P-P PCC means point to point (total 18 points) Pearson correlation coefficient between two time series profiles.

S-S PCC means segment to segment (total 17 segments) Pearson correlation coefficient and the segment (say *i*) value is +1 (-1) if the value at point *i *is less (greater) than that at point *i *+ 1 [53].

Time lag means when aligning the two gene profiles, one of them needs to be shifted relative to the other.

#Edges means the total interactions reconstructed;

#Published means the reconstructed interactions that were previously published;

%Published is the percentage of the published interactions among all the reconstructed interactions;

%GO BP means the percentage of the reconstructed interactions whose genes or gene products pair share a common Gene Ontology (GO) Biological Process (BP) annotation from the SGD GO Slim mapper [46];

*γ *and *R*^2 ^are the power in *P*(*k*) ~ *k*^*γ *^and coefficient of determination returned by the *fit*() function, respectively (see 'Results' – 'Computation Study' section for more details).

As the gold standard or 'true' network is unknown or largely incomplete for this real microarray expression dataset, using criteria such as *recall *and *precision *to evaluate the performance of the reconstruction algorithms is inappropriate and likely to be misleading. There is an emerging tendency recently to take biological context into consideration when dealing with functional genomic data [47-49]. By incorporating biological context information into the data integration process and the network recovery procedure, Myers *et al*. [49] demonstrated that the utilization of such an important source yielded dramatic benefit comparing with their earlier work which only used prior knowledge of gene function but did not particularly exploit biological context. In general, most experiments are designed with the goal of investigating a particular biological process in mind [49]. Consequently, it is both necessary and important to inspect the related biological process information when checking the validity of the predicted interactions in a network, especially for situations where gold standard is not available or incomplete. In this study, for the biological relevance of the predicted edges, we report the percentage of edges whose nodes or genes share a common GO BP annotation from the SGD GO Slim mapper [50]. In general, the probability for two genes or gene products to interact with each other is high if they belong to the same biological process.

For the interactions found by our algorithm, when using the point-to-point Pearson correlation coefficient (P-P PCC) between two time series of gene profiles of 18 time points as the value in the correlation matrix with *N*_*C *_= 101 and *N*_*P *_= 4, 38% of which are found to share the same GO BP annotation when no time lag is used; while 31% are found to share the same GO BP annotation when one time lag is used with *N*_*C *_= 101 and *N*_*P *_= 16. When using the segment-to-segment Pearson correlation coefficient (S-S PCC) over 17 segments, the results are 28% with *N*_*C *_= 91 and *N*_*P *_= 11 and 46% with *N*_*C *_= 91 and *N*_*P *_= 1 for with and without time lag, respectively (see Table 1). This percentage (46%) is comparable to the result (45%) on the same dataset in [51] by using the PCC. Of all the interactions in the network constructed in [51], 3.5% are published interactions in comparison with those (14.08% – 28.33%) in the current study.

## Discussion

In our proposed algorithm, it is required to specify whether a node is a core node or a periphery node. In case of reconstruction of genetic regulatory network, it is not hard to identify transcription regulators from biological knowledge, therefore the core nodes. Consequently, the core and the periphery nodes can be always specified for a set of genes whose networks are to be reconstructed by the proposed algorithm.

We have also mentioned that in our current study the edges in the recovered networks are directionless, i.e., interaction between a pair of nodes is indicated without specifying which node is the source of influence. When more accurate information is needed, the directions of the edges have to be considered. The direction between core and periphery nodes can be always assigned as from the core node to the periphery node since transcription factors always regulate target genes. Several other possible ways to assign the directions for the connections between core and core nodes or periphery and periphery nodes can be considered:

a) compare the rank of node *i *with respect to node *j *and the rank of node *j *with respect to node *i*. Assign the direction of the connection as from the higher ranked node to the lower ranked node. Generally, regulators tend to have more connections and targets tend to have fewer connections. Thus the rank of a regulator with respect to a target tends to be high while the rank of a target with respect to a regulator tends to be low. When there is a tie, a random direction is assigned.

b) for the time-lagged computation, always assign the direction of the connection as from the node without time lag to the node with time lag. This is in accordance with the fact that the expression level of a regulator changes before it can influence its target.

At the same time, we are seeking even better and more efficient ways to improve this method such as specifying neighbor size for each node according to biological knowledge. It is also noted that although the proposed algorithm demonstrated improved performance over the previous one for simulated networks with underlying scale-free property, our algorithm does not directly use any information on the node degree distribution. Therefore, we expect that this algorithm can be applied to the construction of biological networks that are not random.

## Conclusion

A modification of the current algorithm for the scale-free network construction has been proposed and evaluated with two different simulated gene expression datasets and one microarray gene expression dataset. The proposed algorithm performs much better than the original one in recovering the underlying true networks. Compared with previously published experiments using Bayesian Network approaches, our algorithm shows its advantages when the number of samples is small relative to the number of genes, as is the case for most actual biological microarray experiments. The proposed algorithm is expected to be used in reconstruction of biological networks that have underlying scale-free topologies. Besides, as the original algorithm was recently successfully used in gene expression data clustering analysis [43], our improved algorithm hopefully can be incorporated into such clustering algorithm frameworks to derive better clustering results.

## Methods

### Datasets

#### Simulated gene expression data

**Figure 4:**
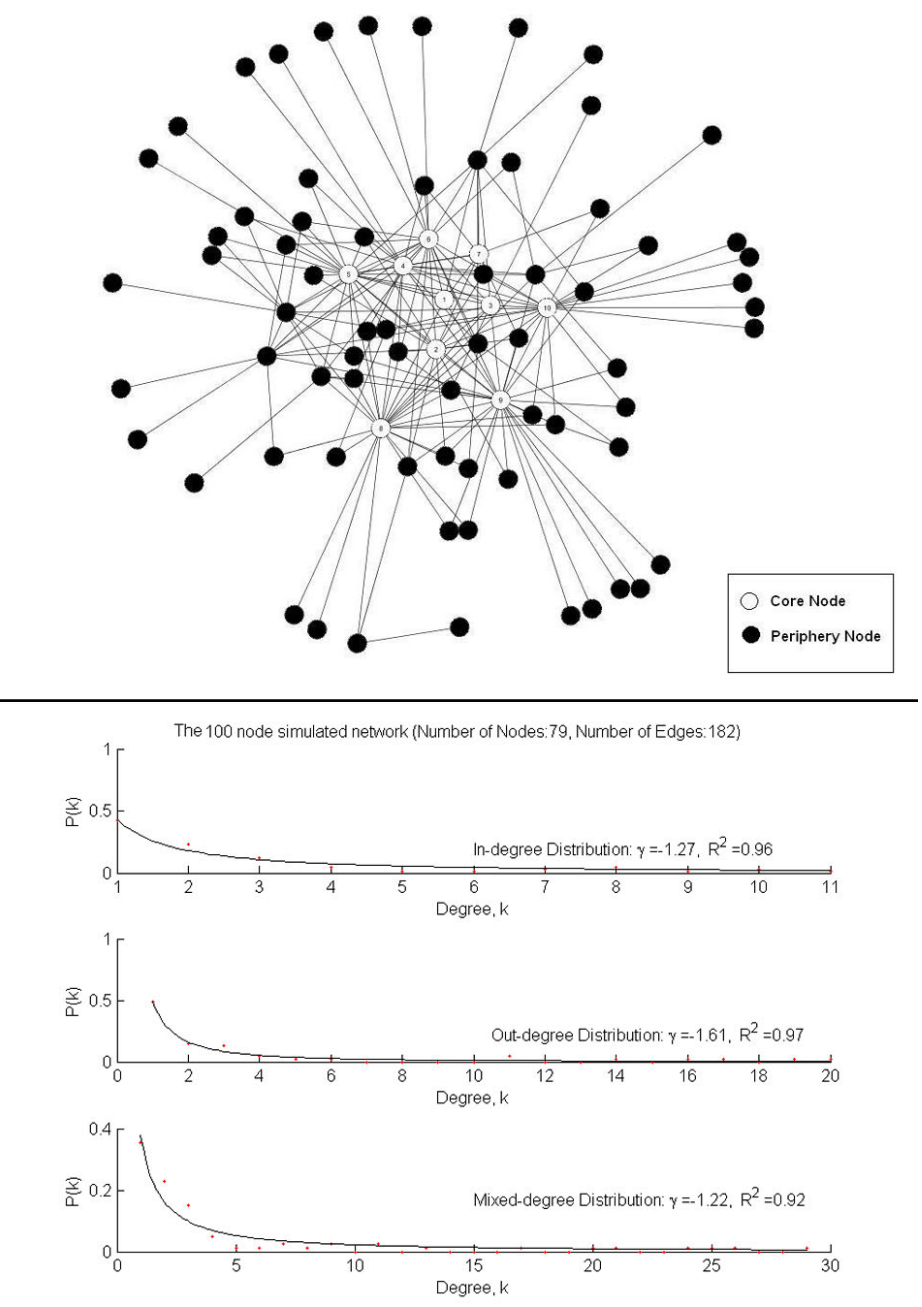
The 100-node simulated network and its node degree distributions. Core nodes are the 10 nodes that form the initial network. Periphery nodes are the remaining nodes that are (preferentially) attached (see 'Methods' – 'Dataset' section for more details).

The underlying scale-free network is a 100-node network constructed by selecting initially 10 core nodes in the network. The connections are made between pairs of these 10 nodes with a pre-specified probability. Either direction for the connection is equally likely. Thus an initial small random network is formed. Then the remaining 90 periphery nodes are added into the network. The nodes to be connected in the existing network with the new coming node are selected preferentially, that is, nodes with higher degree of connectivity will be more likely to be chosen to link to the newly added node. The directions of new connections are more likely (by setting a pre-specified probability) to be from core nodes to periphery nodes. Due to the randomness of the procedure a node might not be connected to any other node in the final network. In the 100-node network, 79 nodes form a large connected component and the others are isolated from this main subnetwork, the number of edges is 182, and the *γ *in the node distribution function (*P*(*k*) ~ *k*^*γ*^) is approximately -1.27 for in-degree, -1.61 for out-degree, and -1.22 for mixed-degree with the Coefficient of Determination *R*^2 ^about 0.96, 0.97 and 0.92, respectively. The network thus can be considered as scale-free. Here, *γ *and *R*^2 ^are computed with the *fit*() function in Matlab. The 100-node simulated network and its degree distributions are illustrated in Figure 4. The 20-node simulated network was constructed in a similar fashion.

With this fixed network topology, the simulated gene profiles are generated following a two-step procedure described in [9]. First, values at each time step are updated by a simple stochastic process:

*Y*_*t*+1 _= *Y*_*t *_+ *A*(*Y*_*t *_- *T*) + *E*

where *Y*_*t *_is a vector representing the expression levels of all genes at time *t*, the matrix *A *represents the regulatory interactions in the simulated network, the vector *T *represents constitutive expression values for each gene, and the vector *E *models the intrinsic biological noise. Second, expression levels are restricted by a floor and ceiling function to range from 0 to 100 (arbitrary units). Expression levels are initialized randomly with values uniformly sampled from this range [9]. By calculating the Pearson correlation coefficients between pairs of these profiles, the correlation matrix is derived. Since the correlation coefficients will be considered in the proposed method, the actual magnitude of the gene expression chosen in the simulated profiles is not essential.

#### Microarray gene expression data

**Figure 5:**
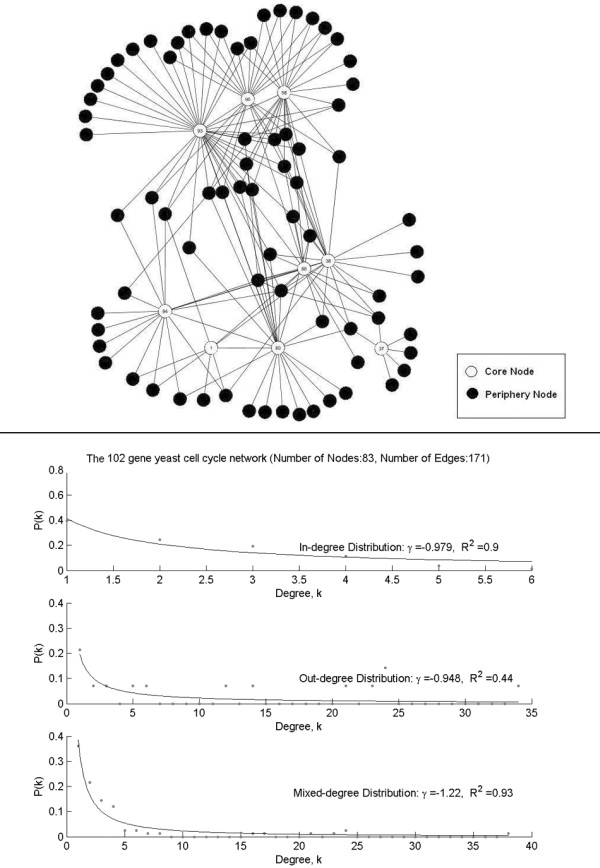
The 102-gene network and its node degree distributions. Core nodes are the 9 transcription factors. Periphery nodes are the remaining non-transcription factors. The edges are obtained from Pathway Studio [45] (see 'Methods' – 'Dataset' section for more details).

The time course profiles for a set of 102 genes are selected from the widely used yeast, *Saccharomyces cerevisiae*, cell cycle microarray data [44]. These microarray experiments were designed to create a comprehensive list of yeast genes whose transcription levels were expressed periodically within the cell cycle. The gene expressions of cell cycle synchronized yeast cultures were collected over 18 time points taken in 7-minute intervals. This time series covers more than two complete cycles of cell division. The 102-gene set includes 9 known transcription regulators and their possible regulation targets [33]. It is highly enriched for known interacting genes involved in the *Saccharomyces *cell cycle. The true edges of the underlying network were provided by the database of Pathway Studio [45], which is based on information derived from PubMed abstracts using natural language search algorithms. If there is confirmative report that gene A and gene B interact with each other, a true edge is then assigned between the pair of genes. For this 102-gene regulatory network, *γ *for in-degree is -0.979 with *R*^2 ^= 0.9, *γ *for out-degree is -0.948 with *R*^2 ^= 0.44, and *γ *for mixed-degree is -1.22 with *R*^2 ^= 0.93. It appears that the distribution for the mixed-degree fits better with the power law distribution. The network and its degree distributions are shown in Figure 5.

#### Algorithm *Symmetric-N*

This algorithm was proposed in [42]. It is presented here for the sake of completeness.

*ConstructedNet *= *Symmetric-N*(*NumNodes*, *N*, *CorrelationMatrix*)

Step 1: for *i *= 1 to *NumNodes*

   *SortedNeighbor *[*i*, 1:*NumNodes *- 1] = *mySort*(*i, CorrelationMatrix*);

Step 2: for *i *= 2 to *NumNodes*

   for *j *= 1 to *i *- 1

      if (*j *is in *SortedNeighbor *[*i*, 1:*N*] and *i *is in *SortedNeighbor *[*j*, 1:*N*])

         *ConstructedNet *[*i, j*] = *ConstructedNet *[*j, i*] = 1;

      otherwise

         *ConstructedNet *[*i, j*] = *ConstructedNet *[*j, i*] = 0;

Here *NumNodes *represents the total number of nodes in the network; *N *the pre-specified number of neighbors; and *CorrelationMatrix *the pre-computed absolute values of the correlation coefficients for all pairs of nodes. The function *mySort*() returns the other nodes in the sorted order in terms of their 'closeness' or correlation with the selected node.

#### Algorithm *Asymmetric-N*

*ConstructedNet *= *Asymmetric-N*(*NumNodes, N*_*C*_, *N*_*P*_, *CorrelationMatrix*)

Step 1: for *i *= 1 to *NumNodes*

   *SortedNeighbor *[*i*, 1:*NumNodes *- 1] = *mySort*(*i, CorrelationMatrix*);

   if (*i *is a core node) *N*_*i *_= *N*_*C*_; otherwise *N*_*i *_= *N*_*P*_;

Step 2: for *i *= 2 to *NumNodes*

   for *j *= 1 to *i *- 1

   if (*j *is in *SortedNeighbor *[*i*, 1:*N*_*i*_] and *i *is in *SortedNeighbor *[*j*, 1:*N*_*j*_])

      *ConstructedNet *[*i, j*] = *ConstructedNet *[*j, i*] = 1;

   Otherwise

      *ConstructedNet *[*i, j*] = *ConstructedNet *[*j, i*] = 0;

#### *fit*() function in Matlab

*fit*() function [52] fits data to model, especially for (non-linear) curve fitting. It was used to fit the data points (dots in Figures 4 and 5) to some power law distributed model (*P*(*k*) ~ *k*^*γ*^). The returns of the function include *γ *and *R*^2 ^for the best fit it finds. We used *fit*(xdata, ydata, 'power1') in which 'power1' is defined as *y *= a*x^*b*^. More details on the function can be found in Additional files 1.

## Authors' contributions

The main framework was formed by GC and YD. GC implemented the algorithm. PL and EA participated in the computation. YD supervised overall project. All authors have read and approved the final manuscript.

## Supplementary Material

Additional file 1 Matlab *fit*() function. The file provides detail information on the usage and the algorithms used for this function. Click here for file
