# Supplementary material for: Rank-based edge reconstruction for scale-free genetic regulatory networks
Source: BMC Bioinformatics. 2008 Jan 31;9:75. doi: 10.1186/1471-2105-9-75 (PMC2275249; doi:10.1186/1471-2105-9-75)
Supplement: Additional file 1 — Matlab fit() function. The file provides detail information on the usage and the algorithms used for this function. [file 1471-2105-9-75-S1.doc]

The Matlab fit() function is to fit model to data. Its syntax is:

cfun = fit(xdata,ydata,*libname*,options)

where xdata and ydata are column vectors data to be fit; libname is the library model from one of the following:

distribution: Probability distribution models;

exponential: One-term and two-term exponential models;

fourier: Fourier polynomial models;

gaussian: Sums of Gaussian models, up to eight terms;

interpolant: Interpolating models, including linear, nearest neighbor, cubic spline, and shape-preserving cubic spline;

polynomial: Polynomial models, up to ninth degree;

power: One-term and two-term power models;

rational: Ratios of polynomial models, up to degree 5 in both the numerator and the denominator;

sin: Sums of sinusoidal models, up to eight terms;

spline: Cubic spline and smoothing spline models.

Matlab cflibhelp() function displays the names, equations, and descriptions of all models in the curve-fitting library.

options is the fit options structure created by fitoptions() function. A typical call to this function is:

options = fitoptions('Method',*method*)

where *method* can be one of the following:

NearestInterpolant: Nearest neighbor interpolation;

LinearInterpolant: Linear interpolation;

PchipInterpolant: Piecewise cubic Hermite interpolation;

CubicSplineInterpolant: Cubic spline interpolation;

SmoothingSpline: Smoothing spline;

LinearLeastSquares: Linear least squares;

NonlinearLeastSquares: Nonlinear least squares.

If the Method field has the value NonlinearLeastSquares, additional fields can be specified in the fit options structure:

Robust: Specifies the robust linear least squares fitting method to be used. Values are 'on', 'off', 'LAR', or 'Bisquare'. The default is 'off'. 'LAR' specifies the least absolute residual method and 'Bisquare' specifies the bisquare weights method. 'on' is equivalent to 'Bisquare', the default method.

Lower: A vector of lower bounds on the coefficients to be fitted. The default value is an empty vector, indicating that the fit is unconstrained by lower bounds. If bounds are specified, the vector length must equal the number of coefficients. Individual unconstrained lower bounds can be specified by -Inf.

Upper: A vector of upper bounds on the coefficients to be fitted. The default value is an empty vector, indicating that the fit is unconstrained by upper bounds. If bounds are specified, the vector length must equal the number of coefficients. Individual unconstrained upper bounds can be specified by Inf.

StartPoint: A vector of initial values for the coefficients. The default value of StartPoint is an empty vector. If the default value is passed to the [fit](http://www.mathworks.com/access/helpdesk/help/toolbox/curvefit/fit.html)() function, starting points for some library models are determined heuristically. For other models, the values are selected uniformly at random on the interval (0,1).

Algorithm: The algorithm used for the fitting procedure. Values are 'Levenberg-Marquardt', 'Gauss-Newton', or 'Trust-Region'. The default is 'Trust-Region'.

DiffMaxChange: The maximum change in coefficients for finite difference gradients. The default is 0.1.

DiffMinChange: The minimum change in coefficients for finite difference gradients. The default is 10^–8^.

Display: Controls the display in the command window. 'notify', the default, displays output only if the fit does not converge. 'final' displays only the final output. 'iter' displays output at each iteration. 'off' displays no output.

MaxFunEvals: The maximum number of evaluations of the model allowed. The default is 600.

MaxIter: The maximum number of iterations allowed for the fit. The default is 400.

TolFun: The termination tolerance on the model value. The default is 10^–6^.

TolX: The termination tolerance on the coefficient values. The default is 10^–6^.

For more complete and detailed references, please view the following webpages:

<http://www.mathworks.com/access/helpdesk/help/toolbox/curvefit/fit.html>

<http://www.mathworks.com/access/helpdesk/help/toolbox/curvefit/fitoptions.html>
